# Maternal and Fetal Effects of Gestational Vitamin D Concentration

**DOI:** 10.3390/healthcare11162325

**Published:** 2023-08-17

**Authors:** Ki-Wook Kwon, Young-Hyeon Lee, Min-Ho Yeo, Sang-Hyun Park, Hye-Ran Kim, Hye-Sook Kim, Kyung-Soo Chang

**Affiliations:** 1 Department of Clinical Laboratory Science, Catholic University of Pusan, Busan 46252, Republic of Korea; kwonkiw@gmail.com (K.-W.K.); dudgus901622@gmail.com (Y.-H.L.); dualsgh136@naver.com (M.-H.Y.); 2 Department of Clinical Laboratory Science, Kyungnam College of Information & Technology, Busan 47011, Republic of Korea; parksh@eagle.kit.ac.kr; 3 Department of Biomedical Laboratory Science, Dong-Eui Institute of Technology, Busan 47230, Republic of Korea; hrkim@dit.ac.kr; 4 Division of International Infectious Diseases Control, Faculty of Pharmaceutical Sciences, Okayama University, Tsushima-Naka, Kita-ku, Okayama 700-8530, Japan; hskim@cc.okayama-u.ac.jp

**Keywords:** mother, vitamin D, fetal bone growth, vitamin D deficiency

## Abstract

Most (90%) vitamin D synthesis occurs in the skin using sunlight (ultraviolet rays), and 10% is obtained through food. Vitamin D is an essential nutrient for skeletal growth and maintenance, cell proliferation and differentiation, and immune function. This study investigated whether maternal serum vitamin D concentrations induce maternofetal effects. Hematological analysis, serological changes, and precision fetal ultrasound findings were analyzed by maternal vitamin D concentration in gestational weeks 22–25 to ascertain direct effects on fetal growth. Bone density–vitamin D concentration correlation was analyzed. No hematologic or serological effect of maternal vitamin D concentration was detected; however, the sexually transmitted infection and cross-infection rates were inversely proportional to maternal vitamin D concentration. No significant correlation between vitamin D concentration and vertebral and femoral BMD was detected. For fetal growth, biparietal diameter, head circumference, abdominal circumference, femur length, and humerus length were analyzed. Humerus (*p* < 0.05) and femur (*p* < 0.001) lengths were higher in the vitamin D-sufficient group than in the vitamin D-deficient group. Vitamin D concentration did not positively affect hematologic changes and bone density; maternal vitamin D concentration essentially affected fetal bone growth. Vitamin D inhibits sexually transmitted infections in mothers and promotes fetal bone growth. Prevention of vitamin D deficiency, supplementation, or outdoor activities is recommended.

## 1. Introduction

Vitamins are classified as water-soluble and fat-soluble nutrients, and vitamin D is a fat-soluble nutrient that enables both skeletal growth and maintenance as well as calcium homeostasis [1]. Vitamin D can be categorized as D_2_ and D_3_, which are abundant in plants and animals, respectively, and D_3_, which plays an important role in human cell proliferation, regulation of cell differentiation, and immune function. Vitamin D deficiency thereof has been implicated in diabetes, obesity, and metabolic syndrome [2]. Mother vitamin D deficiency may contribute prevalence of STDs (sexually transmitted diseases) [3].

**Figure 1:**
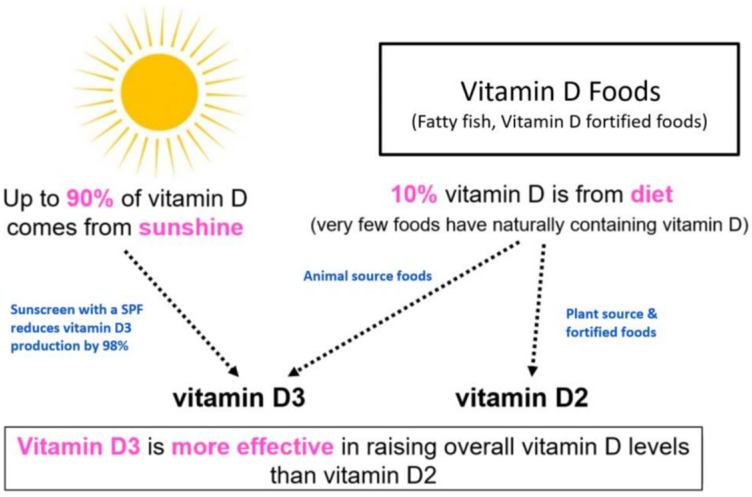
Synthesis and metabolism of Vitamin D.

Vitamin D is absorbed in the body in two ways (Figure 1). As natural vitamin D absorption may be insufficient to attain the requisite vitamin D concentration in the human body, vitamin D absorption through nutrient intake may be needed [4]. The 2015 Korean Dietary Intake Standards recommend daily vitamin D intake of 10 μg (400 IU) and 15 μg (1500 IU) for adults and older adults, respectively [5]. For women aged 19 to 50 years, the United States Food and Drug Administration (USFDA)-recommended dietary allowance (RDA) of vitamin D is 600 IU. The maternal nutritional status affects the overall fetal growth and development in the gestational period, as well as brain development and early bone growth, occurring most actively during the fetal period and infancy [6]. Therefore, the maternal nutritional status, which is directly related to fetal health and growth, needs to be well managed because of its influence on the period from conception through birth to prevent congenital disabilities and pre-eclampsia and enable postnatal cognitive development [7].

In South Korea, the World Health Organization classification criteria for vitamin D levels are used to classify the 25(OH)D_3_ level (deficient, if <20 ng/mL; insufficient, 20–30 ng/mL; and sufficient, 30 ng/mL), which reflects a highly accurate diagnostic measurement of the half-life state [8]. Recently, although studies on maternal and neonatal deficiency or the degree of deficiency of vitamin D have been actively conducted in South Korea [9,10,11], the maternofetal effects of gestational vitamin D deficiency have not been actively investigated.

This study was conducted to determine the need for vitamin D based on an analysis of fetal growth in relation to maternal vitamin D concentration. The target population comprised women in weeks 22 to 25 of gestation whose hematological and serological parameters, as well as the presence of sexually transmitted pathogenic bacteria, were analyzed according to their serum vitamin D concentration. Furthermore, precision ultrasonography was used to analyze the direct effect on fetal bone growth. Moreover, the correlation between maternal bone mineral density (BMD) and maternal vitamin D concentration was analyzed.

## 2. Materials and Methods

### 2.1. Participants and Study Design

This study enrolled 48 expectant mothers with fetuses whose gestational age was 22 to 25 weeks and who visited a mother hospital in Busan between 1 January 2020 and 31 December 2020. The gestational age (weeks) was ascertained based on the measurement of the fetal crown–rump length (CRL) or biparietal diameter (BD) and femur length (FL). Only expectant mothers were invited to participate in the study. Fetuses with congenital anomalies were excluded. The participants included in the final study cohort were mothers who had not experienced any complications, medical diseases, or surgeries and had delivered at full term (after the completion of 37 weeks of gestation). The mothers’ blood tests and fetal ultrasonography were performed. For participants in their 20s and 40s, the BMD was measured.

### 2.2. Vitamin D Level Analysis of the Mother

Immunoserological tests for the quantification of vitamin 25 OH-D_3_ by using an automated immunoassay (Architect i2000sr, Abbott, Chicago, IL, USA) were performed.

### 2.3. Maternal Hematological and Serological Analyses

The hematological tests were performed immediately after blood sample collection into an EDTA tube (BD Vacutainer K2 EDTA), and the blood cell count (WBC, white blood cell; RBC, red blood cell; Hb, hemoglobin, Hct, hematocrit, and Plt, platelet count) was determined by using a hematology analyzer (XP-300 Sysmex Korea Co., Kobe, Japan). Serological tests were performed on a blood sample collected into an SST tube (clot activator and gel, Greiner Bio-One, Frickenhausen, Germany) that was allowed to stand for 10 min and centrifuged (3500 rpm for 15 min) before analysis in a blood biochemical analyzer (TBA-25FR™, Toshiba Technology, Tokyo, Japan). Serum levels of aspartate aminotransferase (AST), alanine aminotransferase (ALT), blood urea nitrogen (BUN), creatinine, total protein, albumin, globulin, glomerular filtration rate (GFR), total bilirubin, and glucose were measured.

### 2.4. Maternal Testing for Sexually Transmitted Diseases

In an early pregnancy test, a cervical cell collection brush is inserted to collect cervical cells, and secretions are collected for Multiplex-PCR analysis (Anyplex™ II STI-12 Detection, Seegene, Seoul, Republic of Korea). The sexually transmitted diseases that are tested for include *Chlamydia trachomatis* (*C. trachomatis*), *Gardnerella vaginalis* (*G. vaginalis*), *Candida albicans* (*C. albicans*), Trichomonas, *Ureaplasma parvum* (*U. parvum*), *Ureaplasma urealyticum* (*U. urealyticum*), *Mycoplasma hominis* (*M. hominis*), *Neisseria gonorrhea* (*N. gonorrhea*), *Treponema pallidum* (*T. pallidum*), *Mycoplasma genitalium* (*M. genitalium*), herpes simplex virus (HSV) type 1, and HSV type 2.

### 2.5. Maternal BMD Measurement

The BMD of the lumbar spine (L1) and femur (neck) was measured using Horizon C (GE Healthcare, Chicago, IL, USA) in 40 women of childbearing age (20–40 years).

### 2.6. Precise Fetal Ultrasound Analysis

The analysis of the fetal body was based on the measurement of BPD, head circumference (HC), abdominal circumference (AC), femur length (FL), humerus length (HL), fetal heart rate (FHR), and estimated fetal weight (EBW) by using fetal biometry ultrasound (Voluson E10, GE Healthcare, Chicago, IL, USA). The ultrasound machine can objectively ascertain abnormalities of the fetal heart through an electronic 4D probe and ultrasound equipment (Voluson E10, GE Healthcare, Chicago, IL, USA). The results of the ultrasound examination were analyzed by the gynecologists themselves to ensure accuracy and reliability.

### 2.7. Statistical Analysis

Statistical analysis was performed in SPSS ver. 27.0 for Windows. A nonparametric technique (Kruskal–Wallis test) was used to determine the correlation between the weekly vitamin D concentration and the measurements of AC, BPD, CRL, and FL; *p* < 0.05 was considered to identify statistically significant differences. Correlations between serum vitamin D concentrations and sexually transmitted pathogens were analyzed using Pearson’s chi-square test.

## 3. Results

### 3.1. Vitamin D Distribution Stratified According to Maternal Age

In this cohort, the 48 mothers were in their 22nd to 25th week of gestation. Regarding the distribution of vitamin D in the blood of pregnant women, 20 patients (41.67%) had vitamin D deficiency, 11 patients (22.97%) had vitamin D deficiency, and 17 patients (35.42%) had vitamin D deficiency (Table S1). Normal levels of vitamin D were obtained from 40 women of childbearing age. The rates of vitamin D deficiency and insufficiency were higher than that of vitamin D sufficiency. In addition, compared to vitamin D concentrations in women of childbearing age, the deficiency group was significantly lower, and the sufficiency group was significantly higher (Table 1).

### 3.2. Hematologic Correlation Stratified According to Serum Vitamin D Concentration

The maternal participants were categorized into vitamin D-deficient, -insufficient, and -sufficient groups based on their blood vitamin D concentration, and the hematologic factors that were possibly affected by vitamin D were investigated. Intergroup comparisons of the maternal vitamin D-related factors showed that the vitamin D-deficient group had WBC (7.80 ± 1.21 × 10^3^/μL), RBC (4.23 ± 0.43 × 10^6^/μL), Hb (12.80 ± 0.77 g/dL), Hct (36.80 ± 2.20%), and Plt (271.00 ± 107.17 × 10^3^/μL); the vitamin D-insufficient group had WBC (7.88 ± 1.47 × 10^3^/μL), RBC (4.21 ± 0.22 × 10^6^/μL), Hb (12.62 ± 0.82 g/dL), Hct (36.74 ± 1.84%), and Plt (286.29 ± 69.51 × 10^3^/μL); and the vitamin D-sufficient group had WBC (8.49 ± 1.15 × 10^3^/μL), RBC (4.10 ± 0.37 × 10^6^/μL), Hb (12.40 ± 1.13 g/dL), Hct (36.13 ± 2.95%), and Plt (279.71 ± 48.30 × 10^3^/μL). Normal levels of hematologic were obtained from 40 women of childbearing age. When the changes in hematological values were analyzed through non-parametric tests, no significant correlation was found with regard to the serum vitamin D concentration (Table 2).

### 3.3. Correlation of Serological Factors with the Serum Vitamin D Concentrations

Normal levels of clinical chemistry were obtained from 40 women of childbearing age. No significant correlation with Vitamin D was found for the changes in serological values in the vitamin D-deficient, -insufficient, and -sufficient groups in the non-parametric tests (Table 3).

### 3.4. Correlation between Serum Vitamin D Concentration and Sexually Transmitted Pathogens

In this cohort, 48 mothers were in the age range of 20–30 and 31–40 years, respectively. Based on their serum vitamin D concentration, 20 (42%), 11 (23%), and 17 (35%) participants were categorized as vitamin D-deficient, -insufficient, and -sufficient, respectively. In the groups with vitamin D deficiency, insufficiency, and sufficiency, the positive and negative rates were 15 (75%) and 5 (25%), 5 (45.5%) and 6 (54.5%), and 6 (35.3%) and 11 (64.7%), respectively (Table 4). Pearson’s chi-square test was performed for vitamin D-deficient, -insufficient, and -sufficient, and STD infection status and *p* = 0.043 was found to be significant. Of the 26 mothers with bacterial infections, the subgroup analysis of participants who were infected with only one type of bacteria revealed that seven, two, and three participants had vitamin D deficiency, insufficiency, and sufficiency, respectively. Among mothers with at least two bacterial cross-infections, five, three, and three had vitamin D deficiency, insufficiency, and sufficiency, respectively. However, three participants in the vitamin D-deficient group had cross-infections of three bacteria (Table 5). The distribution of flora was classified as follows: 16 cases of *G. vaginalis*, 2 cases of *C. albicans*, 14 cases of *U. parvum*, 2 cases of *U. urealyticum*, and 3 cases of *M. hominis*; none of the participants had an HSV type 1 infection (Table 6). However, sexually transmitted infections of clinical significance in gynecology, including *Trichomonas*, *N. gonorrhea*, *T. pallidum*, Herpes, HSV type 2, and *C. trachomatis*, were not detected. There was no correlation between the maternal serum vitamin D concentration and sexually transmitted pathogens (6 species); nonetheless, a correlation of the serum vitamin D concentration with vaginal flora (6 species) was detected.

### 3.5. Correlation between BMD and Serum Vitamin D Concentration in Reproductive Age Women

The age distribution of the 40 women in the reproductive age group is as follows: 8 and 32 women were in the 20- to 30- and 40-year age groups, respectively. Based on the serum vitamin D contributions, participants in the 20–30 years age group were categorized as having vitamin D deficiency (*n* = 7; 87.5%), insufficiency (*n* = 1; 13.5%), and sufficiency (0; 0%) whereas, in the 31–40 years age group, 14 (43%), 11 (34%), and 7 (17%) participants had vitamin D deficiency, insufficiency, and sufficiency, respectively.

**Figure 2:**
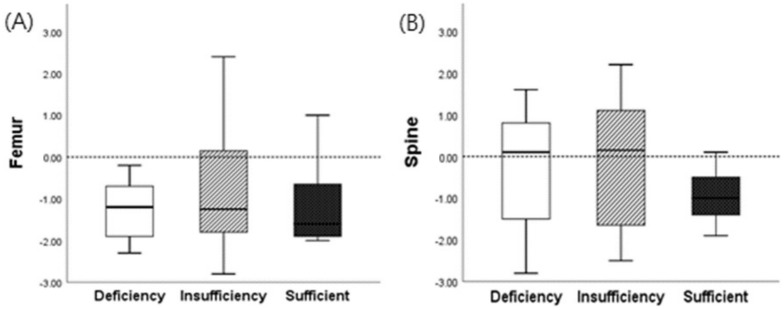
The correlation between vitamin D status and bone mineral density in pregnant women. (**A**) Bone mineral density of the femur according to vitamin D status. (**B**) Bone mineral density of the spine according to vitamin D status.Vitamin D deficiency, insufficiency, and sufficient were defined as 25(OH)D_3_ concentrations <20, 20–30, and >30 ng/mL, respectively. Data are expressed as mean ± standard deviation. Applying the T score: Normal, with a score greater than −1.0; Osteopenia, score within −1.0 and −2.5; Osteoporosis, score below −2.5.

The BMD was determined as T < −2.5 for osteoporosis, −1.0 > T > −2.5 for osteopenia, and T > −1 for normal bone density. To evaluate the correlation between vitamin D and BMD, the bone densities of the spine and femur were measured in reproductive-age women, and the correlation analysis was conducted in the vitamin D-deficient, -insufficient, and -sufficient groups. In groups with vitamin D deficiency, insufficiency, and sufficiency, the BMD values were as follows: spine (L1; 0.15 ± 1.37 g/cm^2^) and femoral neck (−1.20 ± 1.08 g/cm^2^), spine (L1; 0.20 ± 1.52 g/cm^2^) and femoral neck (−1.10 ± 1.45 g/cm^2^), and spine (L1; −0.95 ± 0.73 g/cm^2^) and femoral neck (−1.70 ± 1.05 g/cm^2^), respectively (Table 7 and Figure 2).

In women of reproductive age, 2 (5%), 11 (27.5%), and 27 (67.5%) participants had osteoporosis, osteopenia, and normal BMD, respectively. The femoral neck BMD was found in 1 patient (2.5%) with osteoporosis, 23 patients (57.5%) with osteopenia, and 16 patients (40%) with normal BMD. The correlation between the spinal and femoral BMD was not significant.

### 3.6. Results of Prenatal Ultrasonographic Analysis Stratified by Maternal Serum Vitamin D Levels

The fetal brachiocephalic diameter, HC, AC, femoral bone length, and humeral bone length were measured during the second ultrasound of the mother. The correlation with vitamin D was analyzed based on the standard average value and the length of each part of the body according to the weeks in the standard Korean qualitative fetal values on ultrasound [12,13].

**Figure 3:**
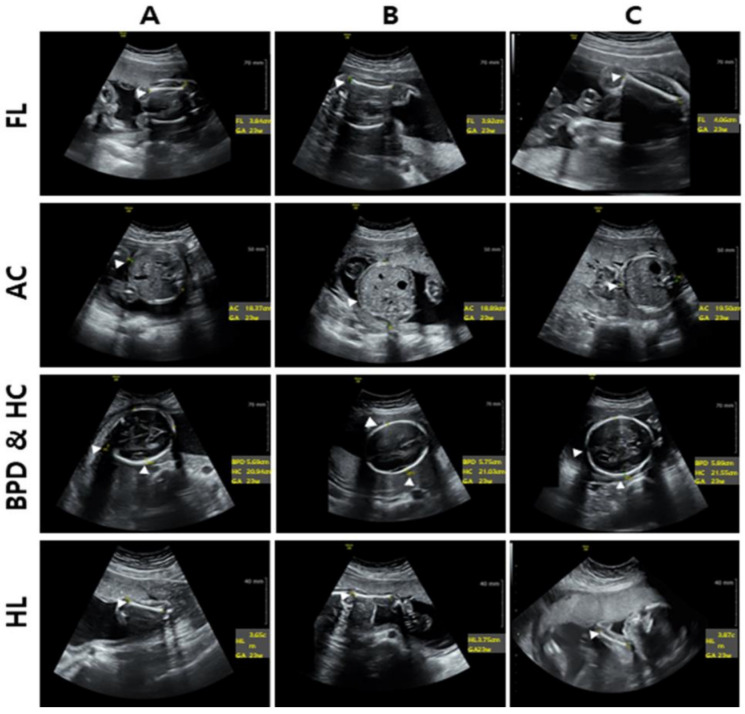
The correlation between vitamin D status and precision of ultrasound imaging in 23-week pregnant women. (**A**) deficient, (**B**) insufficient, (**C**) sufficient were defined as 25(OH)D_3_ concentration <20, 21–30, and >30 ng/mL, respectively. FL, Femur length; BPD, Biparietal diameter; HC, Head circumference; AC, Abdominal circumference; HL, Humerus length.

In the vitamin D-deficient group, the HC was 21.04 ± 0.82 cm, AC was 18.56 ± 0.92 cm, HL was 3.71 ± 0.16 cm, brachial diameter was 5.70 ± 0.34 cm, and FL was 3.92 ± 0.82 cm. In the insufficient group, the HC was 20.44 ± 0.71 cm, AC was 18.37 ± 0.76 cm, HL was 3.64 ± 0.17 cm, the brachial diameter was 5.40 ± 0.37 cm, and FL was 3.84 ± 0.15 cm. In the sufficient group, the HC was 21.58 ± 0.72 cm, AC was 19.08 ± 0.70 cm, HL was 3.86 ± 0.09 cm, hip diameter was 5.71 ± 0.33 cm, and FL was 4.01 ± 0.33 cm. The analysis of vitamin D levels and significance for the five measured items confirmed significance in all four items except BPD. HL and FL showed the highest significance (*p* < 0.001; Table 8 and Figure 3).

## 4. Discussion

In this study, we investigated the relationship between serum vitamin D levels and fetal growth in 48 mothers aged 20 or older who visited a women’s hospital in Busan and underwent a second ultrasound between 22 and 25 weeks of gestation. Vitamin D has an important role in maintaining serum calcium and phosphorus homeostasis by acting on intestinal and bone tissue. Vitamin D that is synthesized in the skin or absorbed from food becomes 25(OH)D in the liver and 1,25(OH)_2_ vitamin D in the kidney [12]. Among the vitamin D metabolites, 25(OH)D has the highest serum concentration, and this concentration reflects the retention of vitamin D in vivo; thus, 25(OH)D is measured during testing [1,13].

In this study, serum 25(OH)D_3_ concentration was measured as an indicator of vitamin D nutritional status in the maternal blood sample, and 35% of mothers had vitamin D deficiency; 23% and 58% of mothers had vitamin D insufficiency. Similar to these results, the number of vitamin D-deficient or -insufficient mothers have been increasing in the United States, Canada, Europe, Australia, and Asia, and this has emerged as a serious health problem [14].

The risk for gestational hypertension, which is one of the most common complications during pregnancy, significantly increases in vitamin D-deficient mothers [15]. In addition, the prevalence of iron deficiency anemia and anemia was high in adolescent girls and adult women with vitamin D deficiency [16]. Anemia is diagnosed when the hemoglobin concentration is 10 g/dL. However, in this study, the results of the analysis of the significance of maternal vitamin D blood level with hemoglobin concentration and hematocrit, which represent anemia, showed no significance. However, the WBC and Plt counts tended to increase in the deficient and sufficient groups as compared to the deficient group, despite no significant difference, and hematological and serological changes due to vitamin D deficiency or deficiency were not significant.

Vitamin D plays a vital role in bone health and immune system regulation through bone mineralization by promoting the absorption of calcium and phosphorus from the intestines and increasing calcium reabsorption in the kidneys to maintain adequate blood calcium and phosphorus levels [17]. Bone mineral density (BMD), a measure of the inorganic mineral content of bone, is a valuable marker in clinical research to assess bone quality [18]. Previous studies of the relationship between vitamin D and bone mineral density have shown a significant effect on vitamin D levels and measurements [19]. However, more recent studies have shown that vitamin D supplementation (low or high doses) does not prevent fractures or falls or have a clinically meaningful effect on bone mineral density [20,21,22].

Women are disproportionately affected by sexually transmitted infections throughout life. Sexually transmitted disease is a highly prevalent vaginal infection that is associated with adverse pregnancy outcomes. Vitamin D influences the immune system and may play a role in sexually transmitted diseases [23]. However, studies with vitamin D have not yet been conducted on the cross-infection of bacteria that cause sexually transmitted diseases in pregnant women.

There are 12 types of sexually transmitted diseases; however, in obstetrics and gynecology, 5 strains are considered clinically significant: *Trichomonas*, *N. gonorrhea*, *T. pallidum*, HSV type 2, and *C. trachomatis.* Seven pathogens, *G. vaginalis*, *C. albicans*, *U. parvum*, *U. urealyticum*, *M. hominis*, HSV type 1, and *M. genitalium*, are considered part of the normal vaginal flora [24]. Analysis of the correlation of vitamin D levels in mothers with single infection and cross-infection with two or three bacteria confirmed a correlation in the deficient and insufficient groups. These results are consistent with those of previous studies, which showed that 25-dihydroxy vitamin D stimulates innate immunity by enhancing bacterial killing, and the results are consistent with previous studies, which showed that adaptive immunity can be modulated to minimize inflammatory and autoimmune diseases. Therefore, vitamin D can affect maternal vaginal health [25].

Vitamin D is converted into an active form when the parathyroid hormone is secreted in response to a decrease in the serum calcium level through mechanisms such as increased intestinal calcium absorption [26]. Maternal vitamin D deficiency, insufficiency, or sufficiency was strongly correlated with the cord blood vitamin D level of the newborn; thus, the mother’s vitamin D intake had a positive effect on the newborn [27,28,29]. In addition, lack of vitamin D and serum calcium, which are important factors for skeletal development during pregnancy or lactation, not only lowers bone mass but also has a decisive effect on the maternal skeletal health, the fetus, and the newborn and infant [30,31,32,33]. Vitamin D and calcium are important for the skeletal health of the mother and fetus: however, studies on the effect of vitamin D or calcium levels and the effect of combined supplementation are scarce [34,35]. This study focused on fetal growth according to the maternal serum vitamin D concentration, and we confirmed that the HC, AC, FL, and HL increased, excluding the subductal transverse diameter. This result is similar to the results of a previous study which found that the maternal vitamin D level is related to the bone density and growth of the newborn and that vitamin D has a beneficial effect on fetal growth [36].

This study confirmed an association between the rate of sexually transmitted infections and fetal femur growth and maternal vitamin D concentration. Vitamin D is essential for the mother’s immune system and the fetus’s growth; therefore, it is important to promote sun exposure and vitamin D intake to prevent vitamin D deficiencies. However, the study did not account for outdoor activities and seasonality, which can affect vitamin D concentrations. Furthermore, the cohort was limited to Busan hospitals and was a small observational study with minimal subject numbers. Therefore, a follow-up study should be conducted with a larger sample size and additional factors such as calcium levels to clarify the relationship between maternal vitamin D concentrations and sexually transmitted infection rates and fetal femur growth in Korea.

## 5. Conclusions

Vitamin D is a nutrient that has an important role in maintaining serum homeostasis. In particular, vitamin D has a positive effect on immunity and growth. There was no significant relationship between maternal vitamin D and hematological and serological parameters. However, the test for normal flora and sexually transmitted diseases showed a decrease in maternal cross-contamination and infection rates. Analysis of the significance of maternal vitamin D and fetal ultrasonographic findings (HC, AC, FL, and HL) showed the high significance of HL (*p* < 0.002) and FL (*p* < 0.001). Thus, maternal vitamin D concentration plays an important role in fetal development and helps increase maternal immunity. Sufficient vitamin D levels are important for maternal health and fetal bone growth, and therefore, it is necessary to encourage adequate vitamin D intake.

## Figures and Tables

**Table 1:** Comparison of serum 25(OH)D_3_ concentrations in women of childbearing age and pregnant women.

	Women of Childbearing Age (*n* = 40)	Pregnant Women (*n* = 48)		
		Deficiency	Insufficiency	Sufficient
Number of women	40 (100%)	20 (41.67%)	11 (22.97%)	17 (35.42%)
25(OH)D_3_ (ng/mL)	20.20 (±10.16)	15.87 (±2.68) **	25.79 (±3.59)	39.58 (±5.80) **

Values are presented as the frequency (proportion). Vitamin D-deficient, -insufficient, and -sufficient categories were defined according to serum 25(OH)D_3_ concentrations <20, 20–30, and >30 ng/mL. Data are expressed as the mean ± standard deviation. Significant differences: ** *p* < 0.01 vs. women of childbearing age group.

**Table 2:** Comparison of hematologic factors in groups and controls by vitamin D concentration in pregnant women.

Hematology		Control	Vitamin D Group			*p*-Value
			Deficiency	Insufficiency	Sufficient	
WBC	10^3^/µL	7.21 (±1.41)	7.80 (±1.21)	7.88 (±1.47)	8.49 (±1.15)	0.776
RBC	10^6^/µL	4.39 (±0.43)	4.23 (±0.43)	4.21 (±0.22))	4.10 (±0.37)	0.801
Hb	g/dL	12.96 (±0.73)	12.80 (±0.77)	12.62 (±0.82)	12.40 (±1.13)	0.713
Ht	%	37.11 (±2.23)	36.80 (±2.20)	36.74 (±1.84)	36.13 (±2.95)	0.936
PLT	10^3^/µL	262.80 (±92.89)	271.00 (±107.17)	286.29 (±69.51)	279.71 (±48.30)	0.711

Values are presented as mean ± standard deviation or number (%). Vitamin D-deficient, -insufficient, and -sufficient status was defined by 25(OH)D_3_ concentrations <20, 21–30, and >30 ng/mL. *p* < 0.05: Significance testing with the hematology test. WBC, white blood cells; RBC, red blood cell; Hb, hemoglobin; Ht, hematocrit; PLT, platelet count.

**Table 3:** Comparison of serum biochemical parameters in groups and controls by vitamin D concentration in pregnant women.

Clinical Chemistry Test		Control	Vitamin D Group			*p*-Value
			Deficiency	Insufficiency	Sufficient	
AST	U/L	18.82 (±3.26)	17.00 (±3.22)	17.04 (±2.84)	19.57 (±3.44)	0.475
ALT	U/L	12.90 (±6.92)	12.00 (±5.89)	12.19 (±5.68)	16.21 (±11.17)	0.707
BUN	mg/dL	8.93 (±3.36)	8.70 (±2.89)	7.95 (±2.25)	7.46 (±1.21)	0.245
CRE	mg/dL	0.63 (±0.11)	0.60 (±0.09)	0.58 (±0.12)	0.66 (±0.12)	0.528
TP	g/dL	7.39 (±0.37)	7.00 (±0.27)	7.10 (±0.45)	7.06 (±0.54)	0.808
Alb	g/dL	4.45 (±0.28)	4.20 (±0.29)	4.34 (±0.23)	4.19 (±0.21)	0.298
Globulin	g/dL	2.82 (±0.27)	2.70 (±0.24)	2.75 (±0.24)	2.85 (±0.43)	0.979
GFR	mL/min/1.73 m^2^	128.39 (±22.19)	127.0 (±20.87)	127.35 (±23.97)	108.81 (±24.36)	0.841

Values are presented as the mean ± standard deviation or frequency (proportion). Vitamin D-deficient, -insufficient, and -sufficient status was defined as 25(OH)D_3_ concentration <20, 21–30, and >30 ng/mL, respectively. *p* < 0.05: Significance testing with hematology test results. AST, aspartate aminotransferase; ALT alanine aminotransferase; BUN, blood urea nitrogen; TP, total protein; Alb, albumin; GFR, glomerular filtration rate.

**Table 4:** Correlation between Vitamin D status and sexually transmitted disease (STD) in pregnant women.

STD	Vitamin D Group		
	Deficiency	Insufficiency	Sufficient
Negative	5 (25.0%)	6 (54.5%)	11 (64.7%)
Positive	15 (75.0%)	5 (45.5%)	6 (35.3%)
Total	20 (100%)	11 (100%)	17 (100%)

Values are presented as the frequency (proportion). Vitamin D-deficient, -insufficient, and -sufficient status was defined as 25(OH)D_3_ concentrations <20, 20–30, and >30 ng/mL, respectively.

**Table 5:** Correlation between vitamin D status and sexually transmitted disease in pregnant women.

Microorganism Infection	Vitamin D Group		
	Deficiency	Insufficiency	Sufficient
Single	7 (46.6%)	2 (40.0%)	3 (50.0%)
2 Cross	5 (33.4%)	3 (60.0%)	3 (50.0%)
3 Cross	3 (20.0%)	0 (0%)	0 (0%)
Total	15 (100%)	5 (100%)	6 (100%)

Values are presented as the frequency (proportion). Vitamin D-deficient, -insufficient, and -sufficient status was defined as 25(OH)D_3_ concentrations <20, 20–30, and >30 ng/mL, respectively. Single infection: Single microorganism infection, 2 Cross infection: Two microorganism infection, and 3 Cross infection: Three microorganism infection.

**Table 6:** Correlation between vitamin D status and single infection and cross infection in pregnant women.

Infection	Microorganism						
		GV	CA	UP	UU	MH	HSV 1
Single Infection	A	3	0	2	0	0	0
	B	3	0	0	0	0	0
	C	1	0	3	0	0	0
2 Cross infection	A	5	0	5	1	0	0
	B	1	0	1	0	1	0
	C	0	0	1	1	0	0
3 Cross infection	A	3	2	2	0	2	0
	B	0	0	0	0	0	0
	C	0	0	0	0	0	0
Total		16	2	14	2	3	0

Values are presented as the frequency (proportion). Vitamin D-deficient, -insufficient, and -sufficient status was defined as 25(OH)D_3_ concentrations <20, 20–30, and >30 ng/mL, respectively. Single infection: Single microorganism infection, 2 Cross infection: Two microorganism infections, and 3 Cross infection: Three microorganism infections. GV, *G. vaginalis*; CA, *C. albicans*; UP, *U. parvum*; UU, *U. urealyticum*; MH, *M. hominis*; HSV, Herpes HSV type 1.

**Table 7:** Correlation between vitamin D status and bone mineral density in pregnant women.

Bone Mineral Density	Vitamin D Group			*p*-Value
	Deficiency	Insufficiency	Sufficient	
Spine (L1)	0.15 (±1.37)	0.20 (±1.52)	−0.95 (±0.73)	0.296
Femur (neck)	−1.20 (±1.08)	−1.10 (±1.45)	−1.70 (±1.05)	0.824

Values are presented as the mean ± standard deviation or as the frequency (proportion). Vitamin D-deficient, -insufficient, and -sufficient status was defined as 25(OH)D_3_ concentrations <20, 20–30, and >30 ng/mL, respectively. *p* < 0.05: Significance cutoff for bone mineral density. Applying the T score: Normal, with a score greater than −1.0; Osteopenia, score within −1.0 and −2.5; Osteoporosis, score below −2.5.

**Table 8:** Correlation between vitamin D status and ultrasound imaging in pregnant women.

Fetus Ultra-Sound Imaging	Vitamin D Group			*p*-Value
	Deficiency	Insufficiency	Sufficient	
HC	21.04 (±0.82)	20.44 (±0.71)	21.58 (±0.72)	0.011
AC	18.56 (±0.92)	18.37 (±0.76)	19.08 (±0.70)	0.11
HL	3.71 (±0.16)	3.64 (±0.17)	3.86 (±0.09)	0.002
BPD	5.70 (±0.34)	5.40 (±0.37)	5.71 (±0.33)	0.638
FL	3.92 (±0.82)	3.84 (±0.15)	4.01 (±0.33)	0.001

Values are presented as the mean ± standard deviation or as the frequency (proportion). Vitamin D-deficient, -insufficient, and -sufficient status was defined as 25(OH)D_3_ concentrations <20, 20–30, and >30 ng/mL, respectively. The size of each part of the fetus is measured in centimeters. *p* < 0.05: Significance test with Precision ultrasound examination. HC, Head circumference; AC, Abdominal circumference; HL, Humerus length; BPD, Biparietal diameter; FL, Femur length.

## Data Availability

Not applicable.

## Supplementary Materials

The following supporting information can be downloaded at: https://www.mdpi.com/article/10.3390/healthcare11162325/s1, Table S1: Pregnancy women characteristics of the distribution.
