# Supplementary material for: Maternal and Fetal Effects of Gestational Vitamin D Concentration
Source: Healthcare (Basel). 2023 Aug 17;11(16):2325. doi: 10.3390/healthcare11162325 (PMC10454327; doi:10.3390/healthcare11162325)
Supplement: Supplementary file 1 [file healthcare-11-02325-s001.zip › healthcare-2507880-supplementary.pdf]

Table S1. Pregnancy women characteristics of the distribution

Independent variables		Percent (%)	Total (%)
Race	a. Asian people	48 (100)	48 (100)
	b. Black people	0	
	c. White people	0	
Urban status[1]	a. Urban	48 (100)	48 (100)
	b. Mountainous	0	
Age groups	a. 20-30	13 (27.08)	48 (100)
	b. 31-41	35 (72.91)	
ABO type	a. A Type	19 (39.58)	48 (100)
	b. B Type	13 (27.08)	
	c. AB Type	7 (14.58)	
	d. O Type	9 (18.75)	
Rh(D) typing	a. Positive	48 (100)	48 (100)
	b. Negative	0	
Smoking status	a. Yes	0	48 (100)
	b. No	48 (100)	
Hepatitis B virus	a. Hbs Ag	12 (25.0)	48 (100)
	b. Hbs Ab	36 (75.0)	
Hepatitis C virus (anti-body)	a. Yes	0	48 (100)
	b. No	48 (100)	
HIV AB	a. Yes	0	48 (100)
	b. No	48 (100)	

[1] Urban status divided by location at urban area
